# Recruitment mechanisms and therapeutic implications of tumor-associated macrophages in the glioma microenvironment

**DOI:** 10.3389/fimmu.2023.1067641

**Published:** 2023-04-20

**Authors:** Xianzhe Zhou, Guishan Jin, Junwen Zhang, Fusheng Liu

**Affiliations:** Brain Tumor Research Center, Beijing Neurosurgical Institute, Beijing Laboratory of Biomedical Materials, Beijing, China

**Keywords:** glioma, glioma-associated microglia/macrophages, recruitment of macrophages, cytokine, tumor microenvironment

## Abstract

As one of the main components of the glioma immune microenvironment, glioma-associated macrophages (GAMs) have increasingly drawn research interest. Primarily comprised of resident microglias and peripherally derived mononuclear macrophages, GAMs are influential in a variety of activities such as tumor cell resistance to chemotherapy and radiotherapy as well as facilitation of glioma pathogenesis. In addition to in-depth research of GAM polarization, study of mechanisms relevant in tumor microenvironment recruitment has gradually increased. Suppression of GAMs at their source is likely to produce superior therapeutic outcomes. Here, we summarize the origin and recruitment mechanism of GAMs, as well as the therapeutic implications of GAM inhibition, to facilitate future glioma-related research and formulation of more effective treatment strategies.

## Introduction

Gliomas are one of the most common adult brain tumors. According to the WHO classification of central nervous system tumors, gliomas are graded as levels 1, 2, 3 or 4 (1). Over recent decades, the treatment of CNS tumors has greatly improved, with therapeutic options currently including surgery, radiotherapy, chemotherapy, and targeted therapy (2, 3). However, median survival time among glioma patients continues to remain low relative to that of patients suffering malignancies such as those of the thyroid or breast. Unfortunately, the median survival of patients suffering glioblastoma (GBM), the most malignant astrocytoma, was recently reported to be approximately 15 months (4, 5).

The tumor immune microenvironment (TME) has long been a focus in oncological research. The TME mainly consists of tumor-associated macrophages, dendritic cells, neutrophils, lymphocytes, astrocytes, and other non-tumor-related cells, and plays a primary role in the promotion of glioma pathogenesis (**Table 1**) (13–16). Glioma-associated macrophages (GAMs) comprise approximately 25% of tumor volume (17) and primarily consist of microglia and macrophages. The presence of both cell types is understood to significantly positively correlate with the malignant progression of glioma (18) and is associated with the cellular acquisition of properties similar to the M2 macrophage phenotype (19, 20). Detailed study of GAM-TME interaction is thus warranted to facilitate development of novel glioma treatment methods and effectively improve glioma patient prognosis.

**Table 1:** Classification and function of immune cells in the immune microenvironment of brain tumors.

Cell Name	Classification	Function	References
**Glioma-Associated Macrophage (GAM)**	M1 Type (Pro-inflammatory) M2 Type (anti-inflammatory)	(1) Inhibit T cell function; (2) Promote glioma epithelial-mesenchymal transition; (3) Promote glioma cell migration	(6, 7)
**Dendritic Cells(DC)**	—	(1) Antigen presentation; (2) Forming tumor immunity	(8)
**Tumor-Associated Neutrophils** **(TAN)**	N1 Type (Anti-tumor) N2 Type (Tumor-promoting)	(1) Inhibit tumor growth; (2) Promote tumor angiogenesis; (3) Promote tumor growth and metastasis	(9, 10)
**Lymphocytes**	Cytotoxic T cells (CD8^+^T Cell) Natural Killer cells (NK Cell)	Antitumor Immune Response	(11, 12)

## The origin and physiological function of glioma-associated macrophages in the tumor microenvironment

**Figure 1:**
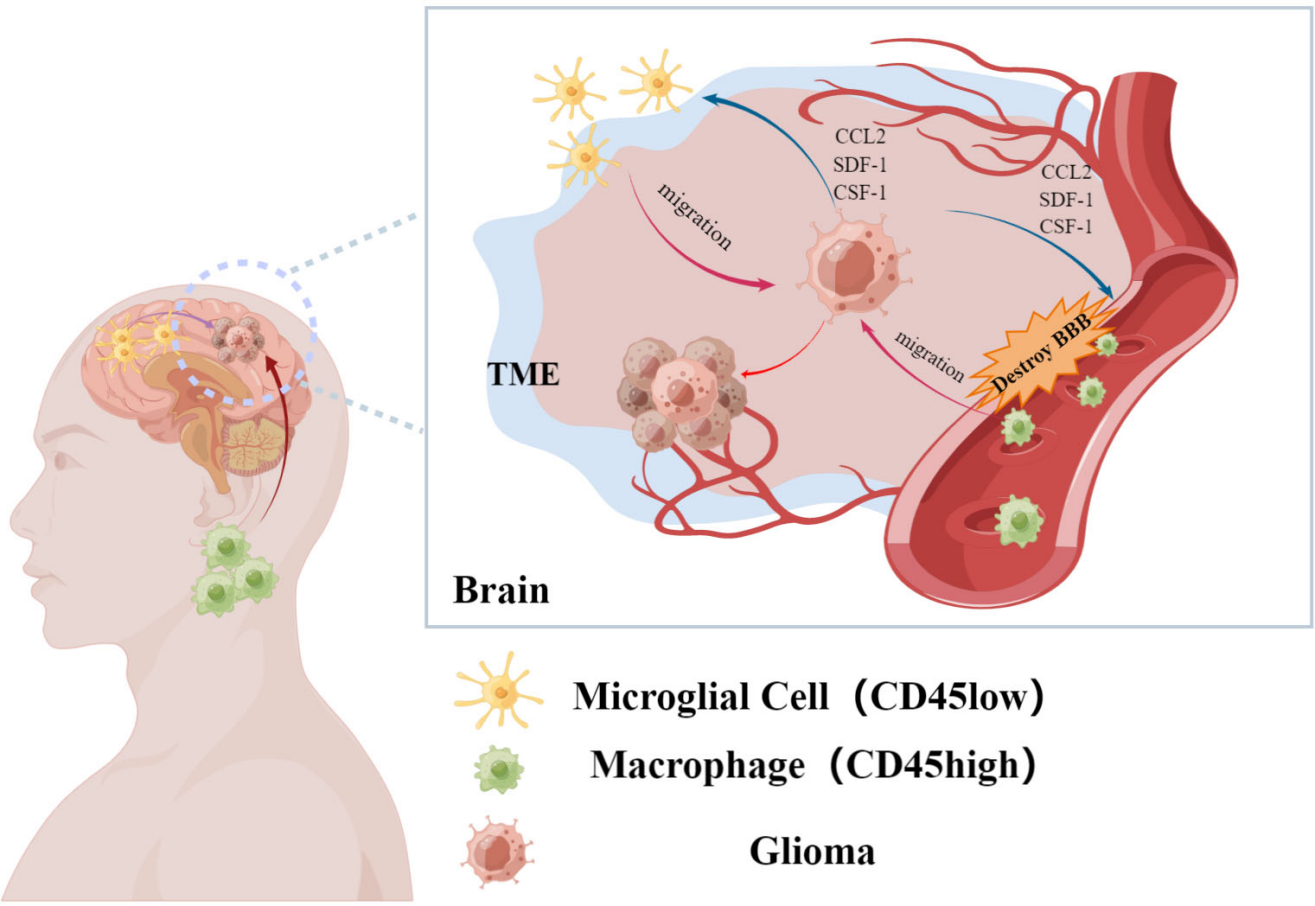
Glioma-associated macrophages are recruited by glioma cells to accumulate in the tumor microenvironment. It mainly includes resident macrophages in the brain and macrophages from peripheral sources, which affect the malignant process of glioma. (By Figdraw).

As the pathogenesis of malignancy progresses, interactions among tumor cells and adjacent tissues result in the formation of the TME, especially in the case of solid malignancy (21, 22). The TME provides a favorable environment for malignant cell growth and enables more effective proliferation as well as resistance to drugs and immunity (23). As the TME develops, a variety of immune cells including tumor-associated macrophages (TAMs), lymphocytes, dendritic cells, and neutrophils are recruited to the vicinity of the tumor (24). However, alterations in molecular interactions within the TME often result in an emergence of many immunosuppressive cells [such as regulatory T cells (25) and TAMs (26)] in and around the tumor. In concert with tumor-promoting molecules, these cells accelerate tumor progression (27, 28). Similarly, GAMs are abundant in and around gliomas (29). Prior studies have confirmed that GAMs in the central nervous system primarily originate from brain-resident microglia and peripherally-derived mononuclear macrophages that enter the central nervous system due to breakdown of the blood-brain barrier (**Figure 1**) (30). While peripherally-derived mononuclear macrophages are mainly distributed in the core region of the tumor, microglia are generally localized in the area surrounding the tumor (17). Of course, this distribution may be related to the recruitment characteristics of the corresponding cytokines. Studies have shown that some cytokines are more likely to recruit macrophages derived from monocytes in peripheral blood (31).

Microglia, derived from yolk sac progenitor cells, appear in the central nervous system at an early stage of ontogeny. The presence of microglia is detected in the brain as early as the ninth embryonic day (32). Traditionally, the distinction between microglia and peripherally-derived mononuclear macrophages is made based on differences in levels of CD45 expression; high levels of CD45 expression (CD45^high^) is considered a characteristic of peripherally-derived cells while low levels of CD45 expression (CD45^low^) is characteristic of microglia (33, 34). However, microglial expression of CD45 was reported to be up-regulated in the TME (35). Distinguishing between the two aforementioned cell subtypes, collectively referred to as GAMs, can thus be challenging. Of course, previous researches have shown that CXCR1^+^/CCR2^-^ can be used for marking microglia, and CXCR1^-^/CCR2^+^ mark monocyte-derived macrophage. And this method is well accepted by the researchers (36). Despite their different origins, both microglia and peripherally-derived mononuclear macrophages play roles in promoting glioma progression (37)

Cytokines promote GAM migration to the site of the glioma and gradually increase the proportion of GAMs within brain tissue from 10-15% to 30-50%, eventually resulting in GAMs becoming the primary component of the glioma TME (20). Under the influence of glioma immune microenvironment, the polarization direction of Gams changed significantly, forming the GAM population dominated by M2 type (38). And as the grade of glioma increased, the proportion of M2 type GAM is also increasing. This type of GAMs produce cytokines such as TGF-β and IL-6, and generally promote tumor progression (39, 40). Such pathologic changes facilitate cellular invasion and angiogenesis, mediate tumor immune evasion, influence T cell infiltration and function, and induce Treg responses, thus significantly promoting malignant progression of glioma (26, 41). Furthermore, massive GAM infiltration of glioma tissue correlates with a poor patient prognosis; reduction of GAM infiltration often facilitates glioma treatment (26, 42). However, the distinction between M1 and M2 GAMs remains unclear. Frequent co-expression of M1 and M2 genes in the same cell suggests that these two subtypes may not be static (43). Furthermore, use of certain drugs (such as rapamycin), decreased M2-type GAM activation, and increased M1-type GAM activation were reported to restore GAM cytotoxic capabilities and result in glioma cell destruction (44). Although inducing changes in the polarization direction of macrophages may yield the greatest therapeutic benefits, due to the complexity of the *in vivo* system, it is difficult to simulate various conditions *in vitro*. Effectively reducing the number of immune microenvironments in glioma may be a more reasonable therapeutic strategy. However, a recent study on Liposomal honokiol (Lip-HNK) indicated that Lip-HNK repolarizes M2 macrophages into M1 phenotype, which effectively enhances the tumor inhibitory ability of GAM. This may be more reasonable and effective than simply inhibiting GAM. At present, Lip-HNK has entered the phase I clinical trial stage for glioma treatment, and various experiments for this drug are being improved step by step, and its further clinical therapeutic effect is also worth our expectation (45).

## GAM recruitment, activation, and polarization

As mentioned above, 10-15% of brain tissue is normally composed of microglia. Effectively regulate the development and physiological functions of the central nervous system. As glioma pathogenesis progresses, weakening of blood-brain barrier function contributes to TME formation and significantly increased the proportion of GAM in TME (46).

**Figure 2:**
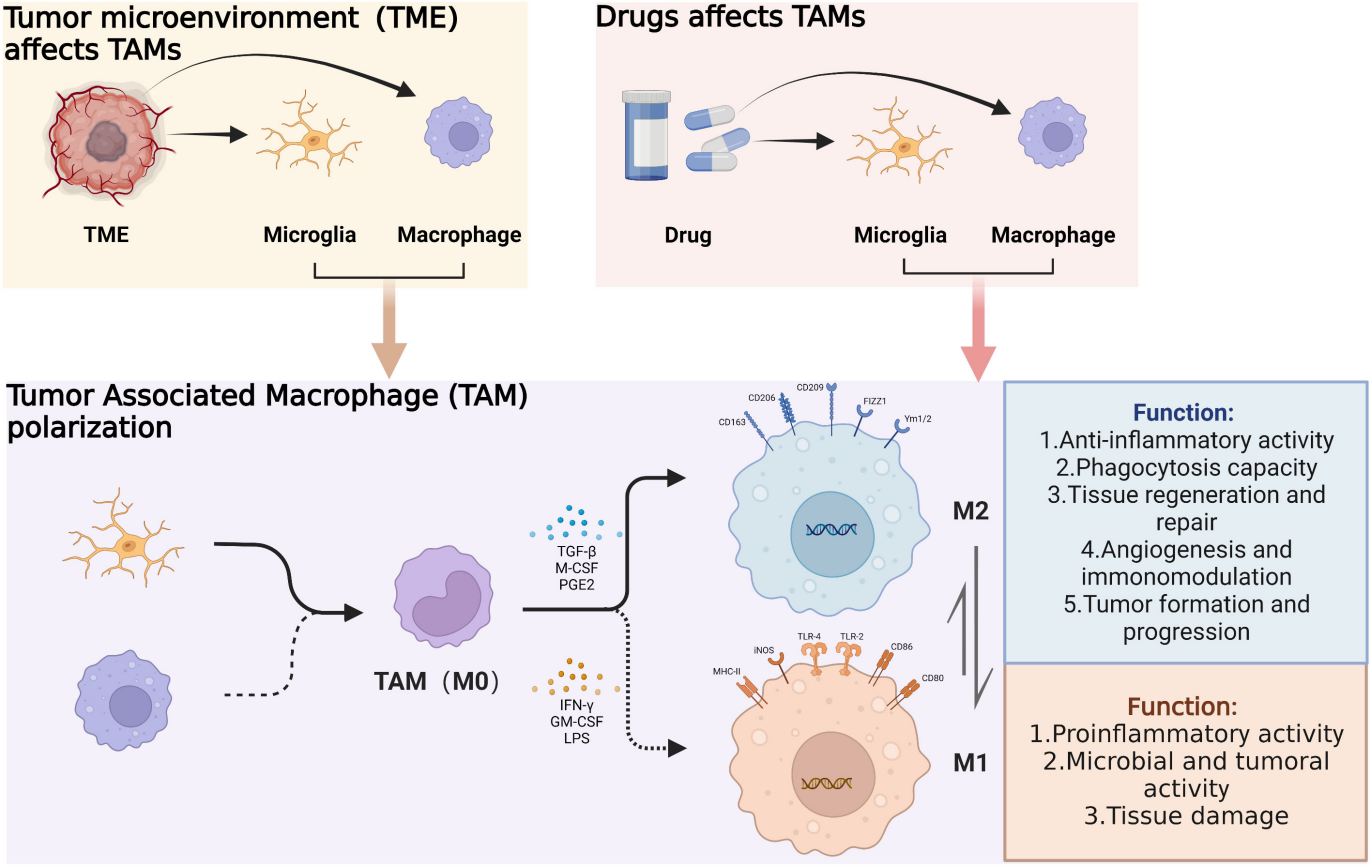
Under the influence of the glioma microenvironment and corresponding drugs, tumor-associated macrophages are recruited toward the tumor. And under the action of certain cytokines, the polarization direction of tumor-associated macrophages changes, resulting in different physiological functions. (By Biorender).

Within the TME, a variety of factors such as cytokines and growth factors activate GAMs and influence cellular polarization. GAMs are generally classified into M0, M1 and M2 subtypes; M2 GAM can be subclassified into functionally different M2a, M2b and M2c cells. Interactions among the aforementioned GAMs phenotypes and glioma cells influence tumor cell invasion and migration, angiogenesis, the tumor-mesenchymal transition as well as immunosuppression (**Figure 2**) (47).

Among the primary factors influencing glioma pathogenesis, GAM accumulation within the TME has long been a research focus. Chemokines, complement receptor ligands and miRNAs all function to recruit macrophages toward the glioma (31, 48). Numerous studies of chemokines have revealed them to play a leading role in the directional migration of microglia. The most extensively studied of these have been CC chemokine ligand 2 (CCL2; MCP-1), stromal cell-derived factor 1 (SDF-1), colony stimulating factor 1 (CSF-1), granulocyte-macrophage colony stimulating factor (GM-CSF), tumor necrosis factor (TNF) and glial cell derived neurotrophic factor (GDNF). Some of these chemokines additionally influence subsequent activation and polarization of GAMs, thereby significantly affecting TME function (49). In this review, major chemokines previously identified to function in macrophage recruitment are summarized to provide foundations for future research directions.

## Major immune cell recruitment factors within the glioma microenvironment

### I. CCL2/CCR2 axis

**Figure 3:**
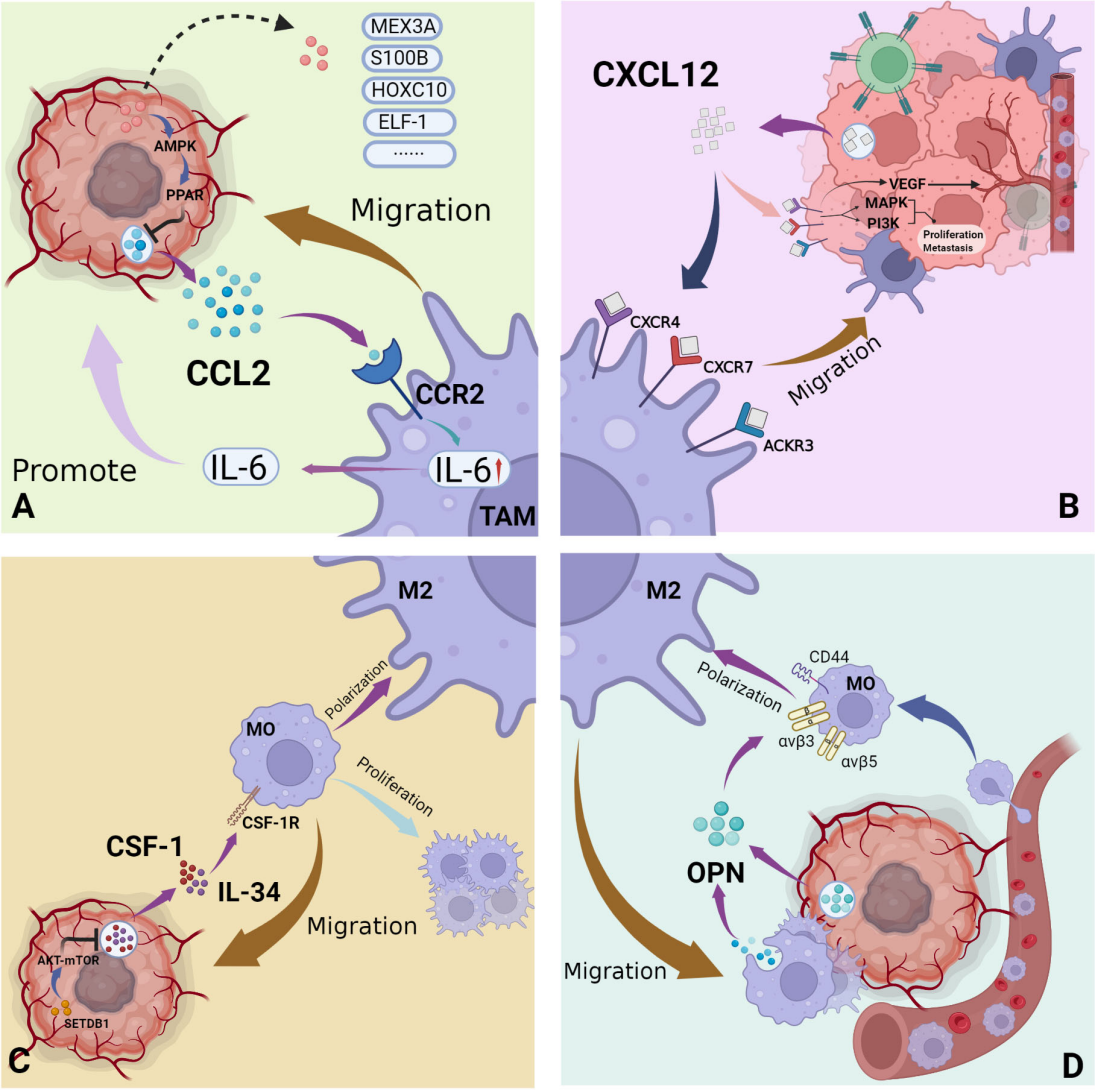
Major recruitment factors of GAMs in the glioma immune microenvironment. **(A)** CCL2 secreted by glioma cells acts on its receptor CCR2 on macrophages, effectively mediating the migration of macrophages to tumor sites and the secretion of tumor-promoting cytokines; **(B)** The three main receptors of CXCL12 secreted by gliomas: CXCR4, CXCR7, ACKR3, exist on the surface of both tumor cells and macrophages. Mediates the recruitment of macrophages, as well as tumor cell proliferation, metastasis and angiogenesis. **(C)** As ubiquitous cytokines, CSF-1 and IL-34 act on the receptor CSF-1R to mediate the recruitment of macrophages and the proliferation and differentiation of macrophages. **(D)** OPN is secreted by tumor cells and macrophages and induces the recruitment and polarization of macrophages. (By Biorender).

Also known as MCP-1, CCL2 plays an important role in tumor growth and macrophage recruitment in the setting of a number of malignancies (**Figure 3A**) (50). For example, breast cancer, gastric cancer, ovarian cancer, etc. (51–53) CCL2 is also present in the central nervous system. A potent chemoattractant, CCL2 is produced by many central nervous system cells such as astrocytes, endothelial cells and microglia (54). Glioma cells also express high levels of CCL2 (17). This was reported to significantly positively correlate with GAM quantity within the glioma TME. However, whether CCL2 directly acts on tumor cells remains unclear. An earlier study of the U87 glioma cell line utilized flow cytometry to confirm that these cells lack CCR2, the CCL2 receptor. Moreover, CCL2 did not appear to markedly influence proliferation or migration of U87 cells (55). A different study of the U251 glioma cell line, however, suggested differently: after CCL2 knockdown, glioma cells exhibited significantly decreased proliferation and migration, and significantly increased apoptotic activity, as compared to control cells (56). Effects of CCL2 on glioma cells thus warrant further clarification.

A number of molecules also influence macrophage infiltration of the central nervous system *via* interactions with CCL2 and thereby affect glioma pathogenesis. Among these, MEX3A expression was found to be significantly increased in the setting of glioma and significantly positively correlated with the degree of malignancy as well as poor prognosis. A likely downstream target of MEX3A, CCL2 is also regulated by this protein. Possible relevant pathways include PPARα/RXRα activation and AMPK signaling (57). Under CCL2 knockout conditions, the tumor-promoting effect of MEX3A was found to be significantly attenuated (56). Homeobox C10 (HOXC10), known to induce angiogenesis *via* VEGFR upregulation, is also highly expressed in the setting of glioma and exerts a similar regulatory effect on CCL2 (58). As HOXC10 knockdown was confirmed to inhibit CCL2 expression, it thus likely plays an important role in CCL2-mediated macrophage recruitment (59). Proteins such as ELF-1 and Notch1 also exert upstream regulatory effects on CCL2, thus affecting macrophage recruitment (60–62).

Upon CCL2 interaction with CCR2, release of IL-6 from microglia is promoted in addition to cellular recruitment. Greater IL-6 levels further increase TME microglia, reduce cytotoxic CD8+ T cells within the TME, and effectively promote malignant proliferation and metastasis (63). Interestingly, a number of studies have reported that CCL-7 (MCP-3) plays a more significant role in GAM infiltration in glioma as compared to CCL2 (64). However, a relatively early publication year of this study, as well as a lack of relevant follow-up research, necessitates verification.

### II. The SDF-1 (CXCL12)-CXCR4/CXCR7 axis

Stromal cell-derived factor-1 (SDF-1), also known as chemokine CXC ligand 12 (CXCL12), is expressed and secreted by a variety of cells including myeloid, endothelial, epithelial and tumor cells (**Figure 3B**) (65). CXCR4, expressed primarily by monocytes and neutrophils in the immune microenvironment, is the primary receptor for CXCL12, but there are others (66). For example, CXCR7 and atypical chemokine receptor 3 (ACKR3) also bind CXCL12 and exert corresponding downstream regulatory effects. Signaling pathways such as the mitogen-activated protein kinase ACCRA (MAPK)/extracellular signal-regulated kinase (ERK) and phosphatidylinositol 3-kinase (PI3K)/protein kinase B (PKB) are activated under the influence of the CXCL12-CXCR4/CXCR7 axis, thus affecting cellular proliferation and metastasis (67, 68). The regulation of angiogenesis by the CXCL12-CXCR4/CXCR7 axis attracts increasing research attention. Studies have reported that the CXCL12/CXCR4 axis effectively upregulates VEGF expression within the TME, thereby promoting angiogenesis. Inhibition of VEGF alone, however, is known to increase CXCR4 expression. Existing experimental treatment methods therefore utilize CXCR4 and VEGF inhibitors in combination have achieved good therapeutic effect (66). The CXCL12-CXCR4/CXCR7 axis also influences resistance to radiotherapy and chemotherapy, immune cell infiltration into the TME and tumor stem cell proliferation. Although the relevant mechanisms of these phenomena are not described in detail in this review, such activity greatly affects malignant pathogenesis (69–73).

In glioma, CXCL12-CXCR4/CXCR7 was reported to be significantly increased. Indeed, increased expression of CXCR4 is considered to be one hallmark of malignant glioblastoma (74). Its ligand CXCL12 was further identified as a potential biomarker of cancer stem cell resistance to radiotherapy, likely *via* induction of autophagy among malignant cells (75, 76). CXCR4 was preferentially expressed in glioma stem cells. As the differentiation degree of glioma increased, the expression level of CXCR4 decreased gradually, instead, the expression level of CXCR7 increased (77, 78). This axis is involved in malignant processes such as tumor angiogenesis, inflammatory response, immunosuppression and reprogramming (79). Recruitment of monocytes is one of the many tumor-promoting effects exerted by CXCR4.

A study of tumor-associated fibroblast (CAF)-induced monocyte migration revealed that CXCL12-supplemented media significantly increased monocyte chemotaxis (80). In glioma, the CXCL12-CXCR4/CXCR7 axis similarly plays a decisive role in macrophage recruitment (77). CXCR4 and CXCR7 are highly expressed on the surface of both microglia and glioma cells (78). As CXCL12 concentration within the TME increases, microglia (mainly M2 macrophages) are increasingly recruited toward the vicinity of the malignancy.

Hypoxia, an essential characteristic of the TME, results in enhanced resistance to damage by tumor cells due to an adaptive modulatory response (81). In the setting of glioma, hypoxia further promotes malignant pathogenesis as well as drug resistance (82). The hypoxic microenvironment upregulates members of the hypoxia-inducible factor (HIF) family, which influence glioma phenotype *via* effects on angiogenesis, cellular resistance to therapy and enhanced metastasis (83). Members of the HIF family also affect macrophage recruitment *via* CXCL12 regulation. An earlier study showed that HIF-1α, as an upstream regulator of CXCL12, effectively influences CXCL12 release (84).While HIF-1α regulates CXCL12, it is also activated by CXCL12 *via* RAS/ERK1-2 and PI3K/AKT signaling (85). In another study of a mouse model of astrocytoma revealed that SDF-1 (CXCL12) effectively promotes GAM recruitment to the vicinity of the malignancy in a concentration-dependent fashion within the hypoxic TME. Co-localization of HIF-1 and CXCL12 by immunofluorescence subsequently complemented the aforementioned findings (86).

In the setting of the hypoxic TME, HIF members play regulatory roles in the CXCL12-CXCR4/CXCR7 axis along with other cytokines such as VEGF and CD26 (87, 88). A 2006 study of the U251 glioma cell line revealed that VEGF increases expression of both SDF-1 and CXCR4 mRNA, thus effectively improving metastatic capabilities (89). In addition, CXCL12 induces macrophage polarization toward the M2 subtype, thus further promoting malignant process (73).

### III. The CSF-1/IL-34-CSF-1R axis

Colony-stimulating factor 1 (CSF-1) and IL-34 are ubiquitous cytokines of great significance to the regulation of monocyte function. Their common ligand, CSF-1R, is a transmembrane tyrosine kinase receptor widely expressed on the surface of cells such as monocytes and marrow-derived macrophages. Ligand-receptor interaction results in effective promotion of monocyte proliferation and differentiation *via* downstream signaling pathways such as JAK-STAT and PI3K/AKT (**Figure 3C**) (90–93).

Expression of CSF-1 and IL-34 in various tumors is significantly increased. In breast cancer, significant upregulation of CSF-1 effectively promotes migration of non-resident macrophages to the TME and induces their polarization toward the M2 subtype (94). In melanoma, the ERK pathway-mediated RUNX1 transcription factor promotes CSF-1R expression, increasing tumor cell survival and malignancy (95).

In normal brain tissue, CSF-1 and IL-34 are respectively secreted by resident microglia and neurons (96). Both cytokines significantly affect microglial development, maturation and function (97). However, in glioma, especially glioblastoma, these cytokines influence tumor progression. While CSF-1R, CSF-1 and IL-34 directly support tumor cell growth, CSF-1, highly expressed by glioma cells, effectively promotes macrophage recruitment and indirectly promotes malignant pathogenesis *via* GAM interactions (98). One study of the SETDB1 enzyme revealed significantly increased expression in glioma and resultant promotion of CSF-1 secretion *via* AKT/mTOR signaling, thereby enhancing macrophage recruitment and polarization (99). Inhibition of CSF-1R signaling *in vivo* was similarly reported to reduce macrophage infiltration of the TME, further validating the roles CSF-1 and IL-34 play in immune cell recruitment (100).

A number of studies have investigated macrophage recruitment by CSF-1. Although administration of anti-CSF-1R therapy to glioma experimental animals exhibited an excellent initial reaction, nearly half of experimental animals eventually developed drug resistance and tumor recurrence. PTEN/PI3K pathway activation and increased levels of IGF-1 were found in the drug-resistant glioma setting. As such, targeting of the above two mechanisms with a combined therapeutic strategy involving CSF-1R inhibition was reported to improve therapeutic outcome (101). Although these findings suggest that CSF-1 and IL-34 are susceptible to regulation, anti-CSF-1R therapy warrants further study for effective use in glioma treatment.

### IV. OPN/SPP1

Osteopontin (OPN), also known as secreted phosphoprotein-1(SPP1), is highly expressed in microglia of the early postnatal brain and injured adults (102). It is an exocrine immunoregulatory protein involved in the inflammatory process, and also expressed in fibroblasts, dendritic cells and macrophages. It is involved in both physiological and pathological processes such as bone formation, osteoarthritis, obesity and Alzheimer’s disease, as well as carcinogenesis and metastasis (103–106). In the central nervous system, OPN effectively monitors acute or chronic injuries in the central nervous system, such as inflammation (102). In various pathological settings, OPN plays roles in mediating inflammation, inducing immune cell proliferation and attracting mature macrophage migration to the vicinity of the lesion (**Figure 3D**) (103, 107, 108).

A component of the extracellular matrix, OPN primarily binds three receptors relevant in the TME including α4β1, αvβ3 and CD44. Activation of corresponding pathways promotes macrophage recruitment, angiogenesis and T cell inhibition, thereby promoting malignant progression (109). Increased expression of OPN in glioma was reported to significantly positively correlate with the degree of malignancy (110). It has been reported that the increased expression of OPN in glioma is significantly positively correlated with the degree of malignancy, and its expression is effective in maintaining the survival and angiogenesis of glioma cells. Furthermore, OPN increases secretion of metalloproteinase-2 (MMP-2), thereby promoting glioma metastasis. OPN also effectively reduces glioma cell sensitivity to the immune system (106, 111). Selective inhibition of OPN expression in glioma was noted to significantly reduce malignant cell proliferation (112).

As previously mentioned, in various diseases, the secreted glycoprotein OPN effectively induces macrophage migration to the lesion site in a dose-dependent manner (104). Recruitment of M0 and M2 (but not M1) macrophages to tumor tissue *via* αvβ5-integrin signaling facilitates a continued increase in OPN secretion and further enhances macrophage recruitment (111). Interestingly, existing literature is inconsistent regarding effects of OPN on macrophage polarization within the TME. Studies focusing on obesity, liver cancer and colitis underscored that apart from promoting macrophage recruitment, OPN effectively induces macrophage polarization to the M2 phenotype *via* the αvβ3 and CD44 receptors, and activates the downstream STAT3/PPARg signaling pathway (103, 113, 114). However, a glioma study reported that despite regulation of macrophage recruitment *via* CD44, OPN does not significantly influence macrophage polarization (111). Mechanisms relevant to the function of OPN in malignancy thus require further study.

OPN offers unique immunotherapeutic prospects. After OPN knockout, significantly decreased levels of M2 macrophages and tumor cell expression of PD-L1 were reported. These phenomena are not due to direct effects of OPN on tumor cells, but rather due to indirect regulatory effects that induce secretion of CSF-1 by macrophages *via* the PI3K/AKT/NF-κB/p65 pathway. After CSF-1R inhibition, anti-PD-L1 treatment in a mouse model of OPN-expressing malignancy was significantly improved (113). However, these findings are limited to liver cancer, and CSF-1R inhibition therapy needs further investigation.

## Significance of macrophage-targeting treatment in the management of glioma

Although glioma treatment has significantly improved over recent decades, surgical resection, adjuvant radiotherapy and temozolomide-based chemotherapy remain gold-standard treatments (115). Median patient survival time, however, has continued to remain relatively low (4). Furthermore, temozolomide treatment was reported to negatively influence myeloid-derived suppressor cells within the glioma immune TME, such as by increasing M2 macrophage phenotypic markers (115). As such, strategies that target GAM recruitment factors in the setting of glioma continue to draw increasing research interest.

Studies have confirmed that anti-CCL2 antibody treatment of glioma effectively reduces immune cell accumulation within the TME, improves the therapeutic effect of temozolomide and prolongs survival among tumor-bearing mice (116). Inhibition of CCL2 was also found to significantly reduce angiogenesis (117). In a study centered on the combination of CCR2 inhibition and PD-1 blocking, the use of a CCR2 antagonist (CCX872) in combination with anti-PD-1 therapy further improved the median survival of tumor-bearing mice (118).

Focus on the therapeutic potential of the CXCL12-CXCR4/CXCR7 axis revealed FTY720, an immunomodulatory drug used in multiple sclerosis treatment, to possess therapeutic activity against various tumors and improving sensitivity to temozolomide therapy (119, 120). Importantly, FTY720 was found to effectively regulate interactions between glioma cells and GAMs primarily *via* promotion of CXCR4 uptake and inhibition of MAPK-mediated IL-6 secretion.

Significant decreases in glioma volume after administration of CSF-1R inhibitors have also been reported (120, 121). As CSF-1R promotes maintenance of malignant cellular characteristics in the setting of glioma *via* ERK1/2 activation, use of the ERK1/2 inhibitor SCH772984 was reported to effectively reduce subsequent malignant progression (122).

Given the role of OPN/SPP1 on macrophage recruitment and promotion of malignancy, OPN inhibition and antagonism of its corresponding receptors (e.g. CD44) are potential therapeutic strategies that warrant investigation in the context of glioma. The proliferative capacity of glioma cells was reported to be significantly reduced in the setting of CD44 knockout and OPN silencing (123). Relevant upstream and downstream regulatory mechanisms similarly warrant detailed study.

## Conclusion

As knowledge concerning glioma pathology has advanced, the role played by GAMs has drawn increasing interest. Although significant progress regarding GAM origin, polarization and function has been made, mechanisms involved in GAM recruitment remain unclear. Here, we review known GAM functions with the aim of facilitating development of future research focused on elucidation of macrophage recruitment mechanisms as well as effective therapeutic strategies.

## Author contributions

XZ and GJ performed conception, data acquisition, data analysis and wrote the manuscript. FL and JZ reviewed and edited the manuscript, secured funding and supervision. All authors contributed to the article and approved the submitted version.
